# Supplementary material for: Adherence to Vaccines in Adult Patients with Immune-Mediated Inflammatory Diseases: A Two-Year Prospective Portuguese Cohort Study
Source: Vaccines (Basel). 2023 Mar 20;11(3):703. doi: 10.3390/vaccines11030703 (PMC10056318; doi:10.3390/vaccines11030703)
Supplement: Supplementary file 1 [file vaccines-11-00703-s001.zip › vaccines-2245941-supplementary.pdf]

Supplementary Table S1 - Quality appraisal using CASP checklist for cohort studies

CASP cohort study checklist	Answer
Did the study address a clearly focused issue?	Yes
Did the study address a clearly focused issue?	Yes
Was the cohort recruited in an acceptable way?	Yes
Was the exposure accurately measured to minimise bias?	Yes
Was the outcome accurately measured to minimise bias?	Yes
Have the authors identified all important confounding factors?	Yes
Have they taken account of the confounding factors in the design and/or analysis?	Yes
Was the follow up of subjects complete enough?	Yes
How precise are the results?	Can’t tell
Do you believe the results?	Yes
Can the results be applied to the local population?	Yes
Do the results of this study fit with other available evidence?	Can’t tell
Does the study have implications for practice?	Yes
